# Webcam-based gaze estimation for computer screen interaction

**DOI:** 10.3389/frobt.2024.1369566

**Published:** 2024-04-02

**Authors:** Lucas Falch, Katrin Solveig Lohan

**Affiliations:** Institute for the Development of Mechatronic Systems EMS, Eastern Switzerland University of Applied Sciences (OST), Buchs, Switzerland

**Keywords:** eye tracking, gaze tracking, webcam based, gaze on screen, gaze estimation, structue from motion

## Abstract

This paper presents a novel webcam-based approach for gaze estimation on computer screens. Utilizing appearance based gaze estimation models, the system provides a method for mapping the gaze vector from the user’s perspective onto the computer screen. Notably, it determines the user’s 3D position in front of the screen, using only a 2D webcam without the need for additional markers or equipment. The study presents a comprehensive comparative analysis, assessing the performance of the proposed method against established eye tracking solutions. This includes a direct comparison with the purpose-built Tobii Eye Tracker 5, a high-end hardware solution, and the webcam-based GazeRecorder software. In experiments replicating head movements, especially those imitating yaw rotations, the study brings to light the inherent difficulties associated with tracking such motions using 2D webcams. This research introduces a solution by integrating Structure from Motion (SfM) into the Convolutional Neural Network (CNN) model. The study’s accomplishments include showcasing the potential for accurate screen gaze tracking with a simple webcam, presenting a novel approach for physical distance computation, and proposing compensation for head movements, laying the groundwork for advancements in real-world gaze estimation scenarios.

## 1 Introduction

Gaze tracking serves as a prevalent technique for comprehending human attention. Its utility extends to gauging users’ attitudes and attention, making it a valuable tool in fields like market research, adaptive information systems, human robot interaction and more recently, entertainment. Despite a reduction in costs for specialized eye tracking equipment, their ubiquity remains limited. Although the idea of using webcams for eye tracking is not new, existing solutions have yet to achieve the level of accuracy and sampling rates found in even basic commercial eye trackers like those from Tobii Group (2023).

Webcam-based eye tracking solutions often provide a gaze vector from the user’s viewpoint, indicating the direction of the person’s gaze. However, these solutions often lack the ability to project this gaze vector into the environment due to the unknown distance between, for instance, a computer screen and the individual. In this study, we present a novel webcam-based gaze tracking approach designed for precise determination of a user’s gaze on a computer screen. Our approach utilizes appearance-based gaze estimation models, like the OpenVino model (OpenVINO, 2023) or a model trained on the ETH-XGaze dataset (hysts on github, 2023), to determine a unit gaze vector. This vector facilitates the calculation of the distance between the user and the computer screen. It is important to note that our approach works with any model providing a gaze vector from the user’s view point. The accuracy of our method is thus contingent on the precision of the model supplying the unit gaze vector. Our approach considers the user’s spatial positioning in front of the screen, allowing us to compute the user’s physical distance from the screen using only a 2D webcam, eliminating the need for additional markers or equipment.

As part of this study, we conduct a comprehensive comparative analysis, evaluating the performance of our proposed method against established eye-tracking solutions. This includes a head-to-head assessment with the purpose-built Tobii Eye Tracker 5, a cutting-edge hardware solution. We also examine the webcam-based GazeRecorder software and provide insights into our choice of this particular software, which will be explained further in the subsequent section.

The organization of the rest of the paper is as follows. In Section 2, the related work is presented while indicating the shortcomings of the existing work. Section 3 outlines the associated methodologies and underlying principles used in the present work. In Section 4 the developed methods are applied and experiments are conducted. A conclusion and directions for possible future work are presented in Section 5.

## 2 Related work

Eye gaze tracking has been a topic of significant research interest for decades, with various techniques and applications developed to capture and analyze the direction and movement of the eyes in order to infer the user’s attention, intention, or interest. There are various techniques available to track human eye gaze. Broadly, two main approaches dominate the field of eye gaze tracking: model-based and appearance-based (Ferhat and Vilariño 2016; Kar and Corcoran 2017).

**Model-based** methods rely on meticulously crafted geometric models of the eye. In corneal reflection techniques, external lighting, often near infra-red (NIR) LEDs, create corneal glints used to extract the eye region and estimate gaze through 2D regression, mapping the vector between pupil center and glint to the corresponding gaze coordinate on the screen (Yoo and Chung, 2005; Zhu and Ji, 2005; Hennessey et al., 2006). Conversely, shape-based methods derive gaze direction from observed eye shapes like pupil centers and iris edges (Chen and Ji, 2008; Hansen and Pece 2005). These models are employed in 3D-based approaches to estimate corneal center, optical and visual axes, and ultimately, gaze coordinates by determining intersections with the scene (Sogo, 2013). Additionally, cross-ratio based methods utilize four LEDs placed at screen corners, reflecting off the cornea to estimate gaze projection on the screen plane (Arar et al., 2016).

However, **appearance-based** methods take a different approach, directly utilizing eye images as input. These methods often employ feature extraction techniques, like face and eye detection, before employing advanced algorithms like CNNs to estimate the point of regard (PoR) (Tan et al., 2002; Sewell and Komogortsev, 2010; Muhammad Usman Ghani and Chaudhry, 2013). For example, Chi et al. Chi et al. (2009) leverage an active infrared light source and a single camera to calculate the gaze and utilize a neural network to compensate for head movements by tracking relative positions between pupil and corneal reflex center. Recent advancements underscore the dynamic nature of this field, with newer methods emerging that do not rely on external lighting and the advent of large-scale datasets has significantly advanced deep learning methodologies by providing millions of annotated samples for training CNNs to map facial attributes and eye images to gaze directions. Zhang et al. (2015) presented the MPIIGaze dataset, where they employed a 3D facial shape model to estimate the 3D poses of detected faces. Their CNN architecture learned the mapping from head poses and eye images to gaze directions in the camera coordinate system, considering the 3D rotation of the head from its coordinate system to the camera’s. Sugano et al. (2014) used a similar approach, training a 3D gaze estimator and performing 3D reconstruction to generate dense training data for eye images. Ground truth 3D gaze directions were provided in the 3D world coordinate system. Krafka et al. (2016) introduced the GazeCapture dataset and trained a CNN for eye tracking. They achieving tracking errors of 1.71 *cm* and 2.53 *cm* on iPhone and tablet devices, respectively. Their end-to-end CNN model for gaze prediction was trained without relying on features such as head pose or eye center location. The dataset was meticulously collected using iPhones and iPads with known camera locations and screen sizes and it is unclear how it performs on different devices, screens or cameras. Another contribution by Zhang et al. (2017) proposed a method for learning a gaze estimator solely from full-face images in an end-to-end manner. They introduced a spatial weights CNN method that leveraged information from the entire face. Additionally, the ETH-XGaze dataset (Zhang et al., 2020), with one million labeled samples, has emerged as a valuable resource for gaze estimation research. Numerous studies have utilized this dataset, with Cai et al. (2021) achieving top-ranking performance on the ETH-XGaze competition leaderboard, attaining an average angular error of 3.11°. Many of these appearance-based method only provide a gaze vector and compare their approach between datasets and gaze vector accuracy without even considering the actual point where a person is looking.

Therefore, there remains a lack of consensus in the literature regarding how to quantify the accuracy of gaze estimation approaches. While it would seem logical to denote accuracy as the error in gaze angle estimation, some studies instead measure it as the Euclidean distance between the estimated point of gaze and the true Point of Regard (PoR). Typically, researchers determine the true and estimated PoR by instructing test subjects to focus on specific points displayed on a screen. The challenge with using Euclidean distance lies in the absence of information regarding the distance between the screen and the user, making it challenging to compare different methods effectively.

In a recent study Heck et al. (2023) analyzed 16 publicly available webcam based gaze estimation software options, offering a comprehensive overview of the techniques employed. The evaluation was performed with a fixed head position and an average user-to-screen distance of 60 cm, as only a subset of the solutions allowed for head movements. In their study, the online eye tracker WebGazer (Papoutsaki et al., 2016) with a mentioned accuracy of 4.06 *cm* detects the pupil in a video frame and uses the location to linearly estimate a gaze coordinate on the screen. In addition the eye is treated as a multi-dimensional feature vector using clmtrackr (Audun, 2023) and histogram equalization similar to Xu et al. (2015). To map the pupil location and eye features onto the screen they use continual self-calibration through user interactions, by assuming that gaze locations on the screen match the coordinates of that interaction. Features and user interactions such as clicks and cursor movements are the inputs for a regularized linear regression model to match eye features to gaze locations. The most accurate webcam-based eye tracking solution observed is GazeRecorder (GazeRecorder, 2023) with an accuracy of 1.75 *cm*, a commercial solution, however details about the gaze estimation method is unknown. Allowing head movements typically results in a decrease in accuracy, even observed in GazeRecorder GazeRecorder (accessed 2023), which experienced a 9% reduction.

2D gaze estimation typically involves formulating a regression problem, where the input image is mapped to a 2-dimensional on-screen gaze location *p* using a regression function *f*(*I*), where *f* represents the regression function and *p* is typically defined on the target screen (Zhang et al., 2017). However, the trained regression function may not be directly applicable to different cameras without addressing differences in projection models. A common assumption in many approaches is that the target screen plane remains fixed in the camera coordinate system, limiting the freedom of camera movement after training, which poses a practical constraint. Many existing methods report only the error in centimeters of the target point without mentioning the distance between the target plane and the user. They also often compare different methods based on specific datasets, overlooking real-world scenarios where users sit in front of a screen and focus on specific points of interest.

In this research project, we aim to overcome this limitation by proposing a method to determine the physical distance between the user and the screen, using only the unit gaze vector provided by many appearance-based methods. In these methods, CNNs are typically trained on labeled datasets, yielding a gaze vector without projecting it onto a surface. In our proposed approach, we will select two trained CNN gaze vector models and provide a methodology to project the gaze vector onto a screen.

## 3 Methodology

### 3.1 Acquisition of gaze vector

**FIGURE 1:**
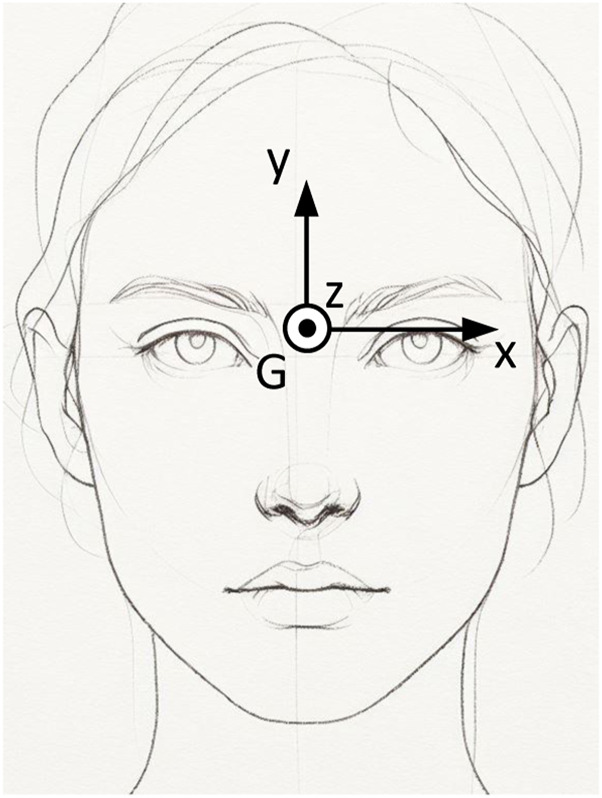
Definition of the gaze coordinate system, denoted as *G*, where the *x*-axis indicates the leftward direction, the *y*-axis points upwards, and the *z*-axis extends towards the screen.

**FIGURE 2:**
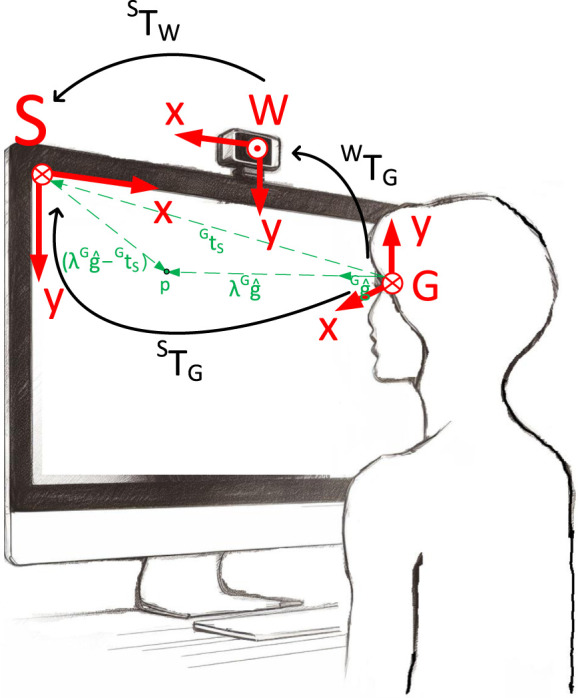
Definition of coordinate systems. *S* is the screen coordinate system, *W* the world or camera coordinate system and *G* the gaze coordinate system additionally depicted in Figure 1. The green vectors illustrate the process of projecting a unit gaze vector onto the screen, as described in detail in Section 3.3.

In this work, we use OpenVINO (Open Visual Inference and Neural Network Optimization) an open-source toolkit developed by Intel for computer vision and deep learning applications. The toolkit includes a range of pre-trained models to simplify the development of computer vision and deep learning applications. The trained models exhibit an optimized format, enabling them to run at maximum efficiency on Intel hardware. Specifically, we use the pre-trained “gaze-estimation-adas-0002” model, which relies on “face-detection-adas-0001,” “head-pose-estimation-adas-0001” and “facial-landmarks-35-adas-0002” models OpenVINO (accessed 2023). First, the face is detected in the webcam video stream. With that, the eye-centers and eyes are detected within the face using “landmarks-regression-retail-0009” model. The detected face is also the input of the head pose estimation model. The gaze estimation model provides a gaze vector corresponding to the direction of a person’s gaze in a Cartesian coordinate system in which the *z*-axis is directed from person’s eyes (mid-point between left and right eyes’ centers) to the camera center, the *y*-axis is vertical, and the *x*-axis is orthogonal to *z* and *y*. This gaze vector is a unit vector and the length is unknown. The subsequent sections will clarify the definition of the coordinate system and visually present them in Figures 1, 2. We further make use of the “pl gaze estimation” model hysts on github (accessed 2023), sourced from a GitHub repository housing trained models derived from various datasets, including MPIIGaze (Zhang et al., 2015), MPIIFaceGaze (Zhang et al., 2017), and ETH-XGaze (Zhang et al., 2020). Specifically, we utilize the model trained on the ETH-XGaze dataset, as showcased in their demo. While numerous gaze vector models are accessible, including offerings from Nvidia (NVIDIA, 2023), we opt for the Intel model, aligning with our hardware infrastructure. Additionally, an online competition assessing the accuracy of gaze vectors for the ETH-XGaze dataset is available (CodaLab, 2023).

### 3.2 Definition of coordinate systems

The OpenVino gaze estimation model’s input is an image of a person and it delivers a unit gaze vector indicating the direction of the person’s gaze. This gaze vector is represented within the coordinate system denoted as *G*, as illustrated in Figures 1, 2. Specifically, the OpenVino model provides this unit gaze vector within the *G* coordinate system, with its location depicted between the eyes in Figure 1. While the exact placement of the gaze coordinate system *G* is not specified on the OpenVino website, its precise location is inconsequential for our method. We determine its position relative to the screen through our proposed approach, as detailed in subsequent sections.

Regarding the ETH-XGaze dataset (Zhang et al., 2018; Zhang et al., 2020), they outline an image normalization procedure where they designate the face center as the midpoint of the four eye corners and two nose corners. Our alignment process for gaze vectors produced by CNN models involves ensuring that the *z*-component of the vector is positive when oriented towards the screen. Additionally, the *x*-component is positive when looking to the left, and the y-component is positive when looking upward. However, this definition is not obligatory for the functionality of our method; only the adjustment of the rotation matrix (Eq. 8) is necessary to align it with the screen coordinate system *S*, and rotation matrix with the world coordinate system *W*, representing the camera coordinate system.

Because the CNN models deliver only a unit vector into the direction where a person is looking, the location of the gaze on the screen is unknown. Therefore, a projection of the gaze vector onto the screen is necessary. In this paper, we provide a methodology to project a gaze vector with unit length onto a screen with unknown distance to the screen.

### 3.3 Projecting gaze unit vector onto computer screen

In the upcoming sections, we will present a novel approach for calculating a homogeneous transformation matrix to determine the position of a user looking at a screen through a webcam. We’ll employ the following notation for the homogeneous transformation matrix (Eq. 1).

TBA=RBAtBA0T1 (1)

TBA is the transformation matrix that transforms a vector in coordinate system *B* to coordinate system *A*. RBA are the unit vectors of the coordinate system *B* presented in coordinate system *A* and tBA is the displacement from *A* to coordinate system *B* presented in coordinate system *A*.

#### 3.3.1 Transformation matrix screen to gaze

The approach to ascertain the user’s distance from the screen and subsequently project the gaze vector onto the computer screen involves the computation of the matrix ^*S*^ *T*_*G*_. The transformation matrix ^*S*^ *T*_*G*_ allows the transformation of the gaze vector ^*G*^ $g\in R^{3}$ from the gaze coordinate system *G* to the screen coordinate system *S* (Eqs 2, 3).

gS=TGS⋅Gg (2)

=RGStGS0T1λGg^ (3)

=RGSλGg^+tGS (4)

The gaze unit vector, denoted as g^G∈R3 , and the scalar λ∈R , project the gaze vector onto the screen. To find *λ* such that the gaze vector g^G intersects with the screen, the scalar product of the screen’s *z*-axis and the vector located on the screen’s plane must be zero (as shown in Eq. 5).

zTGλGg^−tSG=0 (5)

zG=RSG⋅0,0,1T (6)

For a clearer understanding of this equation, please refer to Figure 2, where the dashed green arrowheads represent the vectors involved. The point *p* corresponds to the point on the screen where the person is looking. The vector tSG originates from the transformation matrix TGS , which we will determine in the regression problem discussed in Section 3.3.3. The scalar product with the *z*-coordinate of the screen coordinate system becomes zero when the vector λGg^−tSG lies on the plane of the screen, allowing us to determine the scalar *λ*. Eq. 6 rotates the *z*-axis ([0,0,1]) in screen coordinates to the gaze coordinate system, where RSG follows from the definition of Figure 2. With that we can compute the scaling factor *λ* from Eq. 5.

λ=zTGtSGzTGg^G (7)

As mentioned earlier the “gaze-estimation-adas-0002” model takes as input the two eye images and the head rotation angle and delivers a gaze unit vector, whereby the coordinate system of the gaze vector is rotated so that its *x*/*y*-plane is parallel to the *x*/*y*-plane of the camera. If the webcam is mounted on top of the screen and the *x*/*y*-plane of the webcam coincides with the *x*/*y*-plane of the screen we can determine the rotation matrix RGS .

RGS=−1000−10001 (8)

Therefore, the only unknown is the translation vector tGS∈R3 in Eq. 4. This translation vector is part of the homogeneous transformation matrix, which is determined through a regression analysis (Section 3.3.3).

#### 3.3.2 Calibration process

To gather data for the regression problem, we initiated a calibration process in which a user is presented with points on the screen and instructed to direct their gaze towards these points, which is the standard method. Our approach simplifies the calibration process, involving the presentation of just four calibration points, denoted as ^*S*^ ${\tilde{g}}^{i}\in C$ , on the screen. These calibration points, serve as the user’s focal points. Each point is observed for approximately 2 s, yielding multiple data points for the regression problem from a single point of focus. Transitioning to a new point of focus may introduce a brief lag in gaze redirection, leading to instances where the user does not precisely follow the presented point on the screen. Hence, in the data stream, any erroneous data points, arising when the user gazes at positions different from the presented point, are eliminated prior to the application of the regression model. It is important to note that users were directed to maintain a steady head position during the calibration process, despite not employing a headrest.

#### 3.3.3 Regression problem

With a set C of known calibration points ^*S*^ ${\tilde{g}}^{i}\in C$ displayed on the screen and the gaze vector g^G provided by any gaze vector estimation model, we can determine the translation vector tGS by formulating a regression problem in the following way (Eqs 9–12).

minx∑iC‖Sg~i−giS‖22 (9)

with giS=SRGλiGg^i+tGS (10)

λi=zTG⋅−RGTStGSzTGg^iG (11)

x=tGxS,tGyS,tGzST (12)

The coordinates tGx,y,zS represent the *x*, *y*, and *z* coordinates of the vector tGS . giS is derived in Eq. 4, while *λ* ^*i*^ is obtained from Eq. 7. Given the knowledge of the calibration points’ poses and thus the distances between these points on the screen, we have both a physical reference and a direction provided by the set of gaze vectors ^*G*^ *g* ^*i*^. Leveraging this information, we can determine the 3D location of the gaze coordinate system *G* through the proposed regression problem. Subsequently, we can ascertain the homogeneous transformation matrix TGS and project the unit gaze vector g^G onto the computer screen, as detailed in Section 3.3.

#### 3.3.4 Improve gaze estimation

**FIGURE 3:**
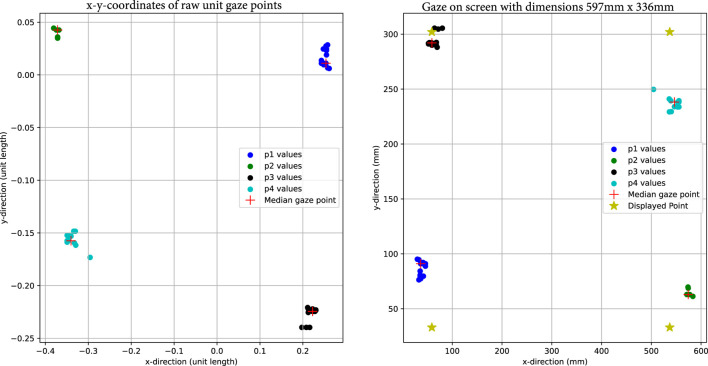
On the left are the already filtered *x* and *y* coordinates of the raw unit gaze vector. Different colors present different calibration points. The red cross presents the median of the noisy gaze vector values. On the right are the raw gaze vectors projected onto the computer screen. The yellow star shows the location, where the points were displayed in the calibration process.

The accuracy of the gaze vector model, in this case, the OpenVino gaze vector, is constrained by inherent limitations. Notably, the accuracy of the gaze vector provided by OpenVino exhibits a Mean Absolute Error (MAE) of 6.95° OpenVINO (accessed 2023). This inaccuracy became apparent in the experiment, where four points forming a rectangular pattern were presented on the screen. Examination of the raw gaze data from the OpenVino model shows that the recorded coordinates do not align with the expected rectangular pattern (Figure 3) that were presented as calibration points on the screen. This occurs because variations in lighting conditions, such as light sources positioned at the side, can result in reflections in the eye, potentially distorting the output.

However, during the calibration process we are able to reduce this inaccuracy, which we will explain next. A homogeneous transformation matrix, incorporating a gaze vector as the translation vector from the calibration, yields an accurate projection for a specific calibration point, albeit limited to that point alone. Consequently, we derive additional transformation matrices from calibration points, each accurate only for its respective calibration point location. Instead the transformation matrix determined in the previous Section 3.3.3 provides a comprehensive estimation across all points. The subsequent equations provide these additional estimations (Eqs 13, 14).

TpS=ItpS0T1 (13)

TGp=RGS−RGTSλGg^0T1 (14)

TGS=TpSTGp (15)

*p* ∈ *P* is the set of calibration points *p* and g^G is the unit gaze vector from a person looking at this point during the calibration process. tpS are the calibration points displayed on the screen during the calibration process. The scaling factor *λ* is computed over Eq. 7 by using tSG from the transformation matrix determined over the regression process. The matrix TGS computed in Eq. 15 transforms the unit gaze vector for this specific point to the screen with zero error.

In total, this procedure provides *n* + 1 transformations TGS , with *n* for the number of calibration points and an additional one determined through the regression problem, providing the distance between the user and the computer screen.

To determine the final gaze point on the screen, we first compute an initial gaze point on the screen through the transformation matrix determined through the regression analysis in Section 3.3.3, which will provide an initial guess. We improve this guess by computing the euclidean to the known calibration points on the screen and choosing as additional transformation matrix, the one closest to this calibration point. As an approximation we define the median between these two gaze estimation points as the final point on the screen.

Additionally, we propose a framework to compensate for head movements, specifically where a user moves to the left or right in front of the screen. To consider these movements we make use of structure from motion.

#### 3.3.5 Structure from motion for head movements

Structure from Motion (SfM) is a powerful technique in computer vision that aims to extract three-dimensional (3D) information from a collection of two-dimensional (2D) frames. One of the fundamental problems in SfM is to simultaneously estimate multiple crucial parameters from a set of point correspondences between two images (Hartley and Zisserman 2004). These parameters include the 3D coordinates of points in space (^*W*^ *p*), the relative motion of cameras (^*W*^ *R*, ^*W*^ *t*), and the intrinsic properties of the cameras (*K*_1_, *K*_2_). The intrinsic properties of the camera are determined over the OpenCV camera calibration library (OpenCV, 2023). For feature matching between consecutive frames we use the OpenVino “facial-landmarks-35-adas-0002” library (Intel Corporation, 2023). With these features we determine the essential matrix *E* and recover the rotation matrix and translation vector between two frames. By triangulating the points we are able to get 3D points in the world/camera coordinate system. However, we do not know the scaling factor, because there is no reference distance in the video stream. Therefore, we formulate a complete regression problem to determine the homogeneous transformation matrix ^*W*^ *T*_*G*_(*t*). Moving the head to the left or to the right shifts the gaze coordinate system *G*. With SfM we are able to determine a unit translation vector of the transformation matrix ^*W*^ *T*_*G*_(*t*) from the world coordinate system *W* to the gaze coordinate system *G*. As mentioned earlier, given the unknown scaling factor, we will derive the gaze vector gS(t) on the screen using the following equations (Eqs 16–18).

gSt=STW⋅WTGt⋅Ggt (16)

=RWStWS0T1RGWμWt^Gt0T1λGg^t (17)

=RWSRGWλGg^t+RWSμWt^Gt+tWS (18)

With μ∈R and *λ* defined in Eq. 7, we compute tSG over the following equation. To enhance readability, we will hereafter omit explicit time dependencies in the variables:

TGS=STWTGW (19)

RGStGS0T1=RWStWS0T1RGWtGW0T1 (20)

=RWSRGWRWStGW+tWS0T1 (21)

With Eqs 19–21 we can compute tSG in the following way (Eqs 22, 23).

tGS=RWStGW+tWS (22)

tSG=−RGTSRWSμWt^G+tWS (23)

^*W*^ ${\hat{t}}_{G}$ is determined over SfM (Section 3.3.5) and with the following regression model (Eqs 24–27) we determine *μ* and tWS .

minx∑iC‖Sg~i−giS‖22 (24)

withgiS=RWSRGiWλiGg^i+RWSμWt^Gi+tWS (25)

λi=zTG⋅−RGTSRWSμWt^Gi+tWSzTGg^iG (26)

x=μ,tWxS,tWyS,tWzST (27)

tWx,y,zS are the *x*, *y*, *z* coordinates of the vector tWS . By solving this regression problem and knowing the parameters in *x* we can compute the transformation matrix TGS in Eq. 19. To collect data, in order to, solve the regression problem we perform the calibration procedure described in Section 3.3.2. The methods presented in this work have been implemented in Python and are publicly available on GitHub (Falch, 2023).

## 4 Experiments and results

### 4.1 Evaluating the validity of the proposed method

In this study, our primary focus is not on evaluating the precision of the OpenVino CNN gaze estimation model or the model trained on the ETH-XGaze dataset. Instead, our goal is to showcase our proposed method, demonstrated on a single user. As our work does not involve the determination of a gaze vector, we utilize the pre-existing OpenVino gaze vector model. The reported accuracy of the OpenVino gaze vector, as per internal assessments, is 6.95° OpenVINO (accessed 2023). In addition we showcase our method on the demo program for gaze estimation, specifically the ETH-XGaze model available on github hysts on github (accessed 2023). Further available models are based on MIIGaze (Zhang et al. 2015), MPIIFaceGaze (Zhang et al., 2017) or any other model available online.

For this demonstration, we engage a user in a simple calibration process. The user is asked to maintain a stable head position and focus on four specific calibration points displayed near the corners of the screen within a rectangle. However, due to the inaccuracy of OpenVino’s CNN model, the raw gaze vector provided by the OpenVino model lacks precision, and the result is not a perfect rectangle (see Figure 3).

To determine the distance between the user and the computer screen and to project a unit gaze vector onto the screen, we utilize the proposed regression model, which yields an estimated transformation matrix denoted as TGS . With this transformation matrix we can now determine the 3D position of the user in front of the computer screen with only a 2D webcam, without additional hardware or markers.

To validate the user’s position in front of the screen, represented by the translation vector of the homogeneous transformation matrix TGS , we conduct physical measurements. This involves determining the distance between the user and the screen and measuring the location from the upper left corner of the screen to the gaze coordinate system, as defined in Figure 2. This verifies our approach and is therefore the first method we know of, computing the actual physical distance, respectively location of a user in front of a computer screen with just four calibration points and without using stereo imaging. Notably, the accuracy of this 3D position is contingent on the precision of the model providing the gaze vector, in our case, the OpenVino model and the model trained on ETH-XGaze. To further validate our method, we calculate the exact vector that the OpenVino model should have delivered using the inverse of the transformation matrix TG−1S=GTS . Employing the transformation matrix ^*G*^ *T*_*S*_, we can then convert the displayed calibration points into the gaze coordinate system, normalizing them in the process. The calculated normalized gaze vectors obtained through this process are then again used as input for the same regression problem, resulting in the same transformation matrix as initially derived, which concludes our validation. By converting the calibration points into the gaze coordinate system, we can assess the accuracy of the applied gaze estimation model by computing the angle between two vectors (Eq. 28).

edeg=cos−1gTGGg~‖G⁡g‖‖Gg~‖ (28)

^*G*^ *g* is the gaze vector provided by the gaze estimation model and ^*G*^ $\tilde{g}$ is the point displayed on the screen transformed into the gaze coordinate system. For this specific trial we get an average error of the four calibration points of 3.3°, which is in this case lower than the reporter 6.95°. However, it is worth noting that the error varies across the field of view, as illustrated in Figure 3. The same holds for the ETH-XGaze dataset, as illustrated in Figure 8 in the paper of Zhang et al. (2020).

### 4.2 Comparison of gaze tracking software

In this section, we conducted a comparative analysis of our method with two established gaze tracking solutions: the Tobii Eye Tracker 5 (Tobii, 2023) and GazeRecorder GazeRecorder (accessed 2023), which is free for non-commercial use, however the used methods and gaze tracking procedure is not available. The reported accuracy on their website is 1°, while Heck et al. (2023) state an accuracy of 1.43° in their review paper. GazeRecorder stands out as a webcam-based gaze tracking system for computer screens, as reported by Heck et al. (2023). The system is the most accurate among webcam-based gaze tracking software. Although, it is important to note that GazeRecorder necessitates a comprehensive calibration process, involving around 30 calibration points.

The Tobii Eye Tracker 5 is purpose-built for tracking user gaze on a computer screen. This hardware is discreetly mounted beneath the screen and relies on a sophisticated combination of infrared cameras and stereo imaging to precisely monitor the user’s gaze. The precise details of its proprietary technique are kept confidential as a closely held company secret.

In this evaluation, we pitted our method against the high-end Tobii hardware and GazeRecorder.

#### 4.2.1 Comparison with no head movements

For this comparison we use the same trial as described in the previous Section 4. Given that both the GazeRecorder software and our method rely on the webcam, we had to conduct an additional trial for GazeRecorder, as the webcam cannot be simultaneously used by both applications. In the experiment, the user was positioned in front of a computer screen at a distance of 800 *mm*. The screen had a resolution of 2560 × 1440 *pixels* and physical dimensions of 597 *mm* × 336 *mm*.

**FIGURE 4:**
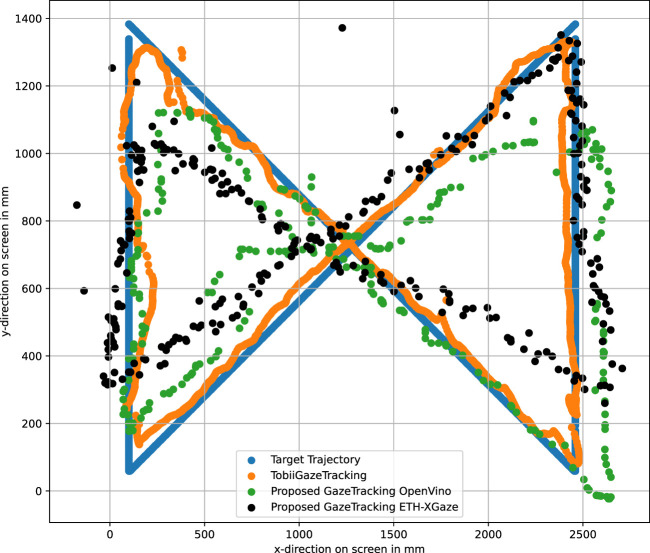
The proposed method is here compared with the Tobii Eye Tracker 5.

To ensure accuracy, we synchronized the recording of both webcam-based gaze tracking solutions with data from the Tobii Eye Tracker 5. In Figure 4, the trajectory of the presented target is depicted in blue, serving as the ground truth. The trajectory recorded by the Tobii Eye Tracker 5 is represented in orange, while our method in combination with the OpenVino model is shown in green and our method in combination with ETH-XGaze dataset is shown in black. Both the OpenVino model and the model trained on the ETH-XGaze dataset yield distinct outputs, both of which serve as inputs in our method for projecting the unit gaze vector onto the screen. This highlights the critical importance of an accurate unit gaze vector for our method. Despite their differences, the overall error along this exemplary trajectory between the two models is comparable (Table 1). It is worth noting that the number of samples collected by the Tobii Eye Tracker 5 surpasses that of the webcam based solutions.

**TABLE 1:** The table presents the RMSE for the three gaze tracking solutions along a predefined trajectory without head movements.

	Tobii eye tracker 5	GazeRecorder	Proposed method	Proposed method
			OpenVino	ETX-Gaze
RMSE	15 *mm*/0.9°	55 *mm*/2.65°	53 *mm*/3.3°	50 *mm*/3.2°

**FIGURE 5:**
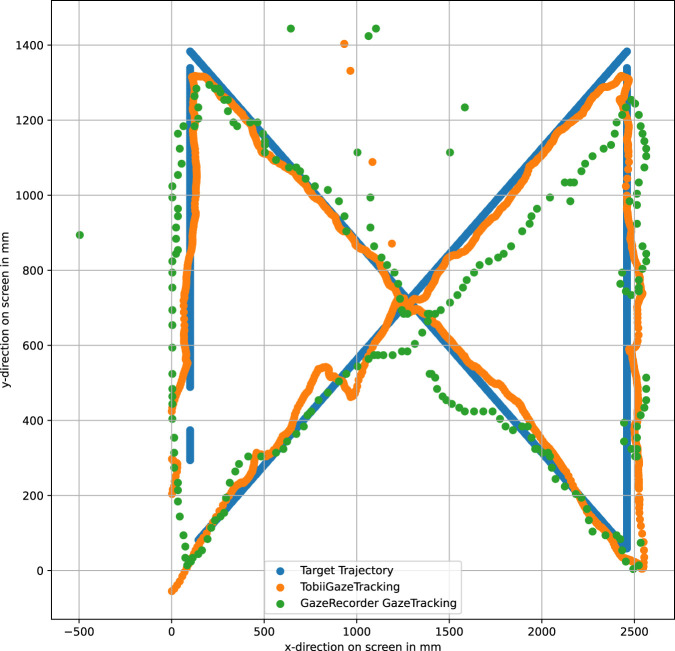
The GazeRecorder is here compared with the Tobii Eye Tracker 5.

In Figure 5, we present a comparison between GazeRecorder, Tobii Eye Tracker 5, and the ground truth. The color scheme in this figure corresponds to that in Figure 4 with the green trajectory for GazeRecorder. Notably, GazeRecorder exhibits a lower sampling rate compared to the Tobii Eye Tracker 5. This lower sampling rate can be attributed to the webcam’s lower frame rate, which affects the rate at which data is captured.

Furthermore, we calculated the Root Mean Squared Error (RMSE) among the three gaze tracking solutions for this trial. The RMSE was computed for the trajectories shown in both Figures 4, 5. The computed RMSE values are presented in Table 1. The RMSE in millimeters represents the discrepancy between the ground truth on the screen and the gaze point provided by each gaze tracking system, at an 800 *mm* screen-user distance. Additionally, the RMSE in degrees measures the angular error between the ground truth gaze vector and the corresponding vector delivered by each of the three gaze tracking systems. It is evident that comparable results were achieved between GazeRecorder, which stands out as one of the most accurate webcam-based gaze tracking solutions, and our proposed method. However, it is essential to note that these solutions still cannot rival the precision of the highly sophisticated Tobii Eye Tracker 5 solution.

For this specific trial, we did not attain the accuracy reported by GazeRecorder and Heck et al. (2023). This deviation could be attributed to the experiments not being conducted in a controlled laboratory environment, with not ideal lightning conditions, potential influences from the user’s attention and gaze tracking behavior when following a trajectory. However, it is essential to highlight that, when computing the RMSE in degrees for this trial, our method yields similar results to GazeRecorder. This serves as an additional indication that our approach to compute a transformation matrix from the gaze coordinate system to the screen is effective, enabling the precise projection of a unit gaze vector onto the screen.

#### 4.2.2 Head movements

In the context of head movements, we differentiate between two types: head movements where the user simply turns their head, corresponding to yaw and pitch movements and head movements where the user moves their head from left to right, or up and down.

For the next experiment, a point is presented at the center of the screen. The user is instructed to focus their gaze on this point while performing head movements: turning their head once to the left, once to the right, and once up and down. This essentially involves yaw and pitch movements. The movements were in the range ±30° for yaw movements and ±25° for pitch movements. The results are presented in Table 2.

**TABLE 2:** The Root Mean Squared Error (RMSE) is calculated to compare the performance of the three gaze tracking solutions.

	Tobii eye tracker 5	GazeRecorder	Proposed method
RMSE	10 *mm*/0.5°	80 *mm*/4.1°	80 *mm*/5.1°

In this particular experiment, a user maintains their gaze on a fixed point while turning their head.

In the following experiment, the user is once more tasked fixing their gaze on a displayed point. However, this time, the user is instructed to refrain from moving their head, but rather, to shift their body to the left and then to the right, all while maintaining their focus on the point. The movement was in a range of ±100 *mm* form the initial position, which was in the middle of the screen at a distance of 800 *mm*. The outcome of this experiment is detailed in Table 3.

**TABLE 3:** The Root Mean Squared Error (RMSE) is calculated to compare the performance of the three gaze tracking solutions.

	Tobii eye tracker 5	GazeRecorder	Proposed method
RMSE	10 *mm*/0.6°	30 *mm*/1.9°	60 *mm*/4°

In this particular experiment, a user moves to the left and then to the right in front of the screen.

The accuracy and precision of our projection depends solely on the gaze vector estimation model used. For instance, the Mean Absolute Error (MAE) of OpenVino is 6.95°, with a standard deviation of 3.58°. Accuracy and precision metrics for models trained on the ETH-XGaze dataset are available on the ETH-XGaze competition website CodaLab (accessed 2023). These initial experiments, though conducted on only one person, already indicate that 2D webcam-based solutions, including GazeRecorder, fail to effectively accommodate precise head movements. These experiments merely serve to highlight the persistent limitations associated with head movements when using 2D webcam-based gaze tracking. This limitation, also recognized by Heck et al. (2023), becomes evident after just one trial. Specifically, lateral movements, such as those to the left or right, are not adequately addressed in any of the trained models. For example, GazeRecorder restricts horizontal head movements by positioning the user centrally and issuing alerts for excessive shifts. In the subsequent section, we delve into the challenges associated with lateral movements, illustrate the issue using the OpenVino model, and propose a solution to address these challenges using SfM.

#### 4.2.3 Applying structure from motion

The proposed method compensates for horizontal head movements in which a user shifts left or right by utilizing SfM to track the user’s movement. To achieve this, we apply the model introduced in Section 3.3.5. In our first experiment using this model, we replicate the conditions detailed in Section 4.2.1, where the user is instructed to follow a target trajectory while keeping their head stationary. The results of this experiment align with those presented in Table 1, showcasing similar outcomes and reinforcing the validity of the approach.

In the subsequent experiment, participants are instructed turning their head to the left and right (yaw-movement). Head movements can introduce subtle changes in the translation vector of TGW since the eyes are not precisely aligned with the head’s axis of rotation. However, it is essential to note that the OpenVino CNN model already compensates for these head movements. Therefore, both yaw and pitch rotations need to be accounted for in the translation vector of TGW .

Furthermore, when a user moves to the left or right without actually performing a yaw head movement, the 2D camera perspective makes it appear as if a yaw movement occurred. This is because the user is filmed from the side, causing the linear movement to mimic a yaw movement from the CNN model’s perspective. These particular movements are not considered in the CNN model’s calculations.

**FIGURE 6:**
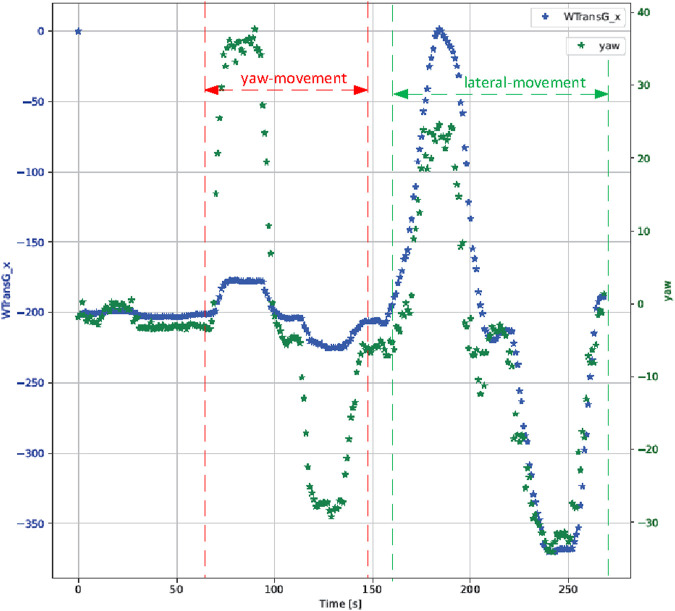
The initial segment represents a yaw movement, while the subsequent segment illustrates a pure lateral shift. The left axis corresponds to the translation vector’s *x*-direction in TGW , while the right axis denotes the yaw angle in degrees.

With the proposed method, which determines the actual location of a person in front of the computer screen, we are able to illustrate the mentioned two scenarios in Figure 6. One movement features a pure yaw movement and another involves lateral head movements to the left and right without head rotation. The visual representation demonstrates that a pure yaw movement leads to a translation in the *x*-direction of the TGW translation vector, as indicated in blue. However, when a user moves laterally, a significant change occurs in the *x*-direction of the translation vector, along with a shift in the yaw angle. This change in the yaw angle happens even when the user does not physically turn their head, a phenomenon attributed to the side-view camera perspective, as previously explained.

To accurately distinguish between these movements, a CNN model should undergo specialized training. This training process should include an additional input: the user’s position in front of the screen, determined through SfM techniques. With existing eye gaze datasets like (Zhang et al., 2020 or Tonsen et al., 2016) we propose to account for lateral head movements and improve webcam based gaze tracking.

## 5 Conclusion and future work

In this study, we presented a novel method for webcam based gaze estimation on a computer screen requiring only four calibration points. Our focus was not on evaluating the precision of any appearance based CNN gaze estimation model but on demonstrating our proposed method on a single user. We presented a methodology to project a gaze vector onto a screen with an unknown distance. The proposed regression model determined a transformation matrix, TGS , allowing the conversion of the gaze vector from the gaze coordinate system to the screen coordinate system.

Physical validation of the user’s position in front of the screen confirmed the soundness of our approach, representing a novel method for determining the physical distance and location of a user without stereo imaging. The method’s accuracy is contingent on the precision of the gaze vector model, here the OpenVINO model and a model trained on the ETH-XGaze dataset.

Comparisons with established gaze-tracking solutions, Tobii Eye Tracker 5 and GazeRecorder, showcased comparable results, indicating the potential efficacy of our method. Further experiments addressed head movements, highlighting the limitations of 2D webcam-based solutions and proposing compensation using SfM techniques.

Future work should focus on refining the proposed method to enhance accuracy, especially in uncontrolled environments. Specialized training of CNN models, incorporating user position via SfM, should be explored using data sets like (Zhang et al., 2020 or Tonsen et al., 2016). The CNN trained with these adjustments should be able to effectively differentiate between lateral movements to the left or right and specific head movements, such as yaw and pitch rotations.

Moreover, there is potential for enhancing sensitivity to cope with varying lighting conditions. The introduction of supplementary filtering mechanisms may aid in bolstering accuracy, particularly in challenging lighting scenarios.

Generally, webcam-based gaze tracking solutions, exhibit a notable sensitivity to varying lighting conditions. It is essential to underscore that the overall accuracy of our proposed method is intrinsically tied to the precision of the raw gaze vector generated by the gaze estimation model. In this study we have conclusively demonstrated that webcam-based solutions, while promising, still cannot attain the level of accuracy achieved by sophisticated gaze tracking hardware, which frequently leverages advanced technologies such as stereo vision and infrared cameras. Consequently, the pursuit of further research endeavors is imperative in order to bridge the existing gap and elevate webcam-based solutions to a level of accuracy comparable to that of purpose-built eye tracking hardware.

## Data Availability

Publicly available gaze estimation models were analyzed in this study. The implementation of the concept presented in this paper can be found here: https://github.com/FalchLucas/WebCamGazeEstimation.
